# Multimedia-based training on Internet platforms improves surgical performance: a randomized controlled trial

**DOI:** 10.1007/s00464-012-2672-y

**Published:** 2013-03-09

**Authors:** Carolina Pape-Koehler, Marc Immenroth, Stefan Sauerland, Rolf Lefering, Cornelia Lindlohr, Jens Toaspern, Markus Heiss

**Affiliations:** 1 Department for Abdominal, Vascular, and Transplant Surgery, Cologne Merheim Medical Center, University of Witten/Herdecke, Ostmerheimerstrasse 200, 51109 Köln (Cologne), Germany; 2 Ethicon Endo-Surgery Europe, Norderstedt, Germany; 3 Institute for Research in Operative Medicine (IFOM), University of Witten/Herdecke, Cologne, Germany; 4 Institute for Quality and Efficiency in Healthcare, Cologne (IQWiG), Cologne, Germany

**Keywords:** Internet platforms, Multimedia-based training, Webop, Surgical training, Pelvi-Trainer

## Abstract

**Background:**

Surgical procedures are complex motion sequences that require a high level of preparation, training, and concentration. In recent years, Internet platforms providing surgical content have been established. Used as a surgical training method, the effect of multimedia-based training on practical surgical skills has not yet been evaluated. This study aimed to evaluate the effect of multimedia-based training on surgical performance.

**Methods:**

A 2 × 2 factorial, randomized controlled trial with a pre- and posttest design was used to test the effect of multimedia-based training in addition to or without practical training on 70 participants in four groups defined by the intervention used: multimedia-based training, practical training, and combination training (multimedia-based training + practical training) or no training (control group). The pre- and posttest consisted of a laparoscopic cholecystectomy in a Pelvi-Trainer and was video recorded, encoded, and saved on DVDs. These were evaluated by blinded raters using a modified objective structured assessment of technical skills (OSATS). The main evaluation criterion was the difference in OSATS score between the pre- and posttest (ΔOSATS) results in terms of a task-specific checklist (procedural steps scored as correct or incorrect).

**Results:**

The groups were homogeneous in terms of demographic parameters, surgical experience, and pretest OSATS scores. The ΔOSATS results were highest in the multimedia-based training group (4.7 ± 3.3; *p* < 0.001). The practical training group achieved 2.5 ± 4.3 (*p* = 0.028), whereas the combination training group achieved 4.6 ± 3.5 (*p* < 0.001), and the control group achieved 0.8 ± 2.9 (*p* = 0.294).

**Conclusion:**

Multimedia-based training improved surgical performance significantly and thus could be considered a reasonable tool for inclusion in surgical curricula.

Surgical procedures are complex motion sequences that require a high level of preparation, training, and concentration. To improve surgical skills, various training possibilities such as virtual reality (VR) training and practical training in lab training classes are used [1, 2]. Although the value of these classes is undisputable, they are cost intensive, time consuming, and bonded to schedules and locations [3, 4].

Unfortunately, not many alternatives exist. Most often, a surgical intern refers to a surgical manual to study and prepare for a scheduled procedure. These surgical manuals describe the procedure with illustrations and texts. One disadvantage of these surgical manuals is their tendency not to be up-to-date due to publishing procedures, which is a relevant problem of all print media in a continuously developing field such as surgery.

As in various other scientific fields, the Internet has become an alternative to print media, offering many alternatives and opportunities. For surgical educators, the Internet offers the possibility of standardizing general surgical trainings and assessments and the opportunity to develop national and international collaborations [5].

In recent years, several Internet platforms have been established that provide surgical know-how in different formats as well as the use of different media and material. They all attempt to offer the latest actual practice. The disadvantages with most of these platforms are heterogeneous content, unknown benefit of the didactic method, and non-evidence-based content [6].

One advantage of the Internet is the use of multimedia. The multimedia approach uses different media at the same time to display certain content. The media work together. The media can be text, graphics, audio, animation, video, data, and the like. An example of multimedia is a Web site with information about the composer Mozart that includes text, an audio file sampling of his music, and perhaps even a video of a concert [7].

The use of various media takes advantage of the different channels of perception [8]. Evidence in the literature proves that multimedia-driven learning has advantages in medical fields wherein an understanding of complex temporal and spatial events plays an important role [9]. Multimedia modules about aortic valve replacement have shown better educational value than print media with the same content for students studying heart surgery [10].

However, in addition to the conventionally used methods, a modern surgical curriculum requires cost- and time-effective training methods as well as the implementation of new didactic methods and material. A new pedagogic paradigm is required [11]. Therefore, it seemed necessary to evaluate the benefit of a multimedia-based platform in surgical education (www.webop.de) [12]: an Internet platform that combines these aspects with the basic background of the mental training method, visualizing nodal points.

Mental training is a cognitive training method taught by mental trainers that includes imagining a movement repeatedly. Findings have shown that mental training increases surgical performance by using operation primers (manuals describing the surgery in nodal points) [13, 14].

The current study tested whether use of the multimedia-based Internet platform (www.webop.de) together with the operation primer, either in combination with practical training or no practical training, improves learning success compared with practical training or no training for participants with little laparoscopic experience. Learning success was defined as improved surgical performance in completing a laparoscopic cholecystectomy in a Pelvi-Trainer.

The main questions of our study were as follows:

1. What is the effect of multimedia-based training on surgical performance?
2. What is the effect of practical training on surgical performance?

## Materials and methods

### Trial design

The randomized controlled trial (RCT) in this study was created using a pre- and posttest design. It was conducted as a 2 × 2 factorial study with four intervention groups (multimedia-based training, practical training, and combination training using either multimedia-based + practical training or no training [control group]) and blinded assessment of training results. Recruitment and follow-up evaluation of participants were performed from February 2009 until August 2009. The study was approved by the ethics review committee of the University of Witten/Herdecke and considered noncritical.

### Study settings

The study took place at the Campus Merheim, University of Witten-Herdecke, Cologne, Germany, which featured a sufficient number of training facilities. Data were collected and analyzed at the Institute for Research in Operative Medicine, University of Witten-Herdecke, Cologne, Germany.

### Study participants

Eligible participants were medical doctors (MDs) participating in surgical fellowships at hospitals in Cologne within a 30-km radius and medical students in their final year at the University of Witten/Herdecke and Cologne University who fulfilled the inclusion criteria. For recruitment of participants, these hospitals and universities were contacted and given written information about the study and a questionnaire (Table 1). They were asked to send back the completed questionnaire. Based on the responses received from the questionnaire, we selected the subjects and invited them to participate in the study. The information provided by the questionnaires was used later to evaluate possible differences in the test groups.

**Table 1:** Questionnaire

Demographic questions
1. Age
2. Sex
3. Date of approbation
Medical career
1. Passed state exams
2. Apprenticeship before medical school
3. Start of surgical fellowship
4. Discontinuation of surgical fellowship
5. Surgical fellow
Surgical experience
6. No. of assisted laparoscopic surgeries
7. No. of self-performed laparoscopic surgeries
8. No. of assisted laparoscopic cholecystectomies
9. No. of self-performed laparoscopic cholecystectomies
10 Attendance of a laparoscopic training course
General practical ability
11. Experience in two-dimensional PC games
12. Ability to eat with sticks
13. Ability to sew a button

To reduce heterogeneity regarding surgical experience, we defined the following inclusion criteria:

- A minimum of one assisted laparoscopic cholecystectomy
- A maximum of seven self-performed laparoscopic cholecystectomies

Subjects who had already completed a surgical fellowship in surgery or had previously attended a laparoscopic training course were excluded from the study.

### Activity areas

#### Pelvi-Trainer

Pelvi-Trainers were used for pretesting, posttesting, and practical training. The Pelvi-Trainer is composed of a plastic housing containing a pork liver and gallbladder. This training dummy has been well evaluated and meets the criteria for simulating a surgical procedure. It offers great resemblance in terms of fidelity, organ properties, organ reaction, interactivity, and sensory feedback.

The Pelvi-Trainer consists of the Pelvi-Trainer itself (Firma Storz, Tuttlingen, Germany), a laparoscopic unit, and a high-frequency (HF) unit for electrocoagulation. Pelvi-Trainers simulate an abdomen in which surgeries can be reproduced in a realistic manner. The same laparoscopic instruments including an HF unit for electrocoagulation are used in the operating room (OR). The picture data recorded by the laparoscopic camera are converted to a monitor.

In the current study, the camera was guided by camera assistants, who exclusively moved the camera when told to do so by the operating participant. Laparoscopic cholecystectomies were performed on pork livers with an intact gallbladder. The pork livers were purchased from a slaughterhouse in the vicinity.

#### Multimedia-based training

**Fig. 1:**
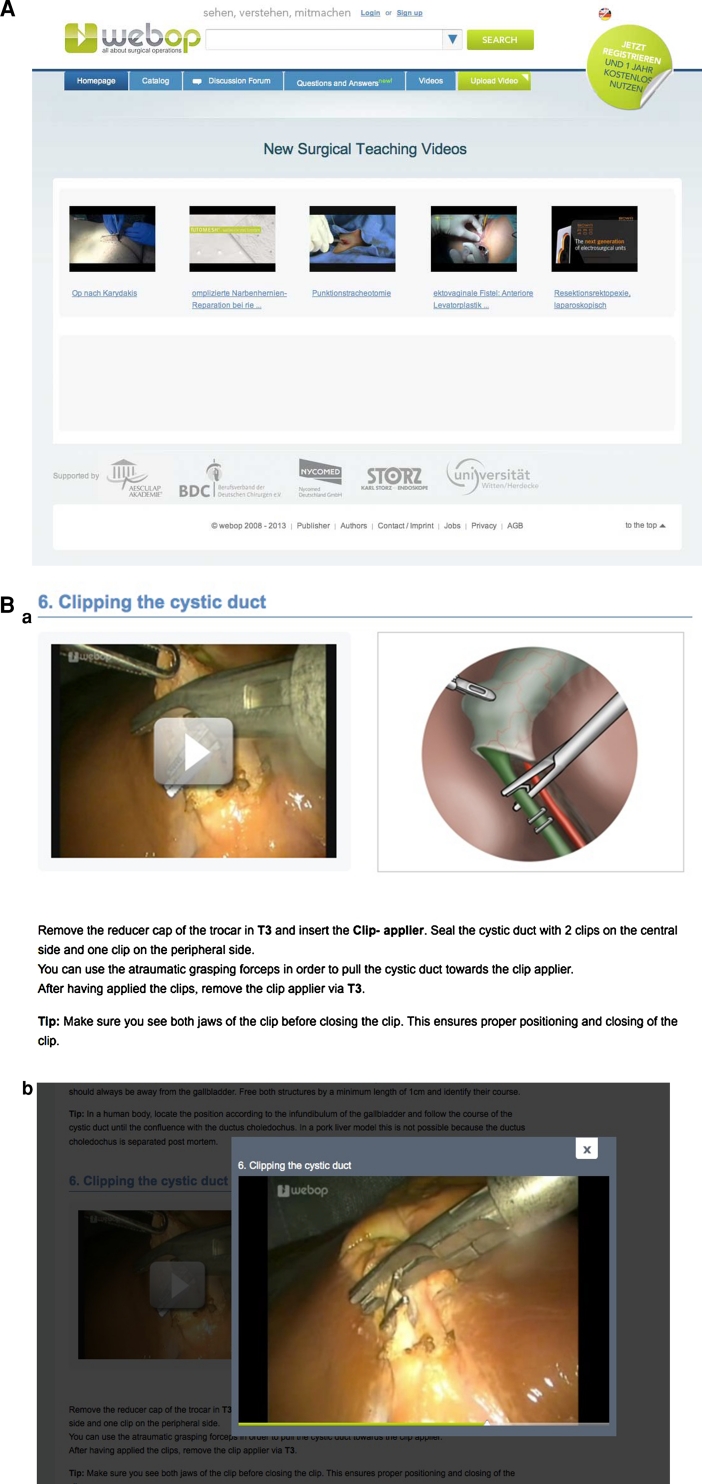
**A** Homepage of www.webop.de. **B** (*a*) On www.webop.de, one procedural step is explained in a combination of text, illustration, and video. The trainee sees procedural step 6 (Clipping the cystic duct) of the chapter laparoscopic cholecystectomy in the Pelvi-Trainer (http://www.webop.de/surgeries/58?locale=en). (*b*) Use of the “start” icon starts the video explanation

The activity area consisted of a personal computer (PC) and a print version of an operation primer. The PC was used for the Webop chapter *Laparoscopic Cholecystectomy in the Pelvi-Trainer*, which was specifically produced for this study (to view it, follow the link http://www.webop.de/surgeries/58?locale=en. On www.webop.de (Fig. 1A), the surgical procedure is shown divided into procedural steps. Each procedural step is typically described in a combination of text, illustration, and video, including explanations of how to perform the procedural step as well as hints on how to avoid mistakes (Fig. 1B).

The videos are composed with explanations. In addition, the participant is able to watch the entire video of the surgery nonstop. Typical Webop chapters consist of additional sections such as surgical anatomy, perioperative management, complications, and evidence. These sections were eliminated in the chapter produced for this study because they had no effect on our objective.

The operation primer provided at the activity area was produced especially for this study following a model by Immenroth et al. [13, 14]. A characteristic of this operation primer is its display of a surgery subdivided into so-called nodal points. Each nodal point gives the instruction for what to do in both text and photographs.

### Randomization

Four participants were invited to each appointment. After the baseline procedure (pretest), they were randomized by lot. Each participant drew an opaque envelope from a box containing one of four different instructions corresponding to the study groups. If there were fewer than four people at one time, randomization took place in the same manner. Enrollment in the study, camera assistance, and evaluation were blinded.

### Participant flow

**Fig. 2:**
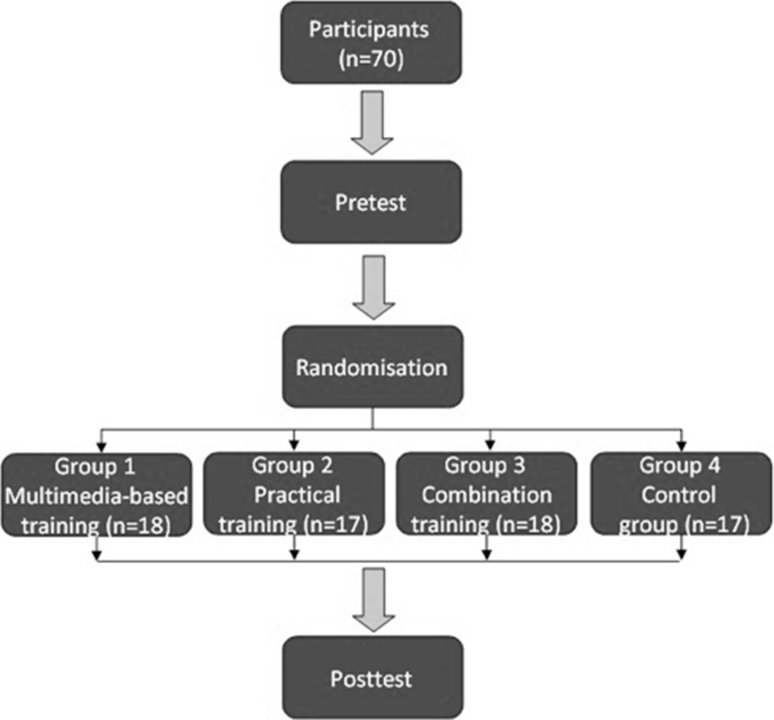
Participant flow

The four participants were registered, and each signed an informed consent. Each experiment consisted of a baseline test (pretest) and an intervention (training: multimedia-based, practical, combination, or none) on day 1 and a follow-up test (posttest) on day 2 (Fig. 2). The pre- and posttest consisted of a laparoscopic cholecystectomy in the Pelvi-Trainer. Before the pretest, all the participants received a standardized explanation of the Pelvi-Trainer and the provided instruments as well as a short instruction for the task they were to perform. During the pre- and posttest, the participants did not receive any advice or instruction.

After the pretest, randomization took place in the manner described earlier. After randomization, each participant was guided to the activity area of the intervention to which he or she was randomized. The participants then received the standardized instruction of the procedure they were to perform, and the 2-h training period began. The interventions are described in the next section.

After the training period, the participants were sent home after they had signed an agreement not use any information channel to improve their knowledge in laparoscopic cholecystectomy. On day 2, 24 h after the pretest, the participants completed the posttest under the same conditions as the pretest.

### Interventions

#### Training modules

##### Group 1 (multimedia-based training)

The participants in this group were led to the activity area (multimedia-based training) after they had completed the pretest. They were briefed to watch the chapter, Laparoscopic Cholecystectomy in the Pelvi-Trainer, on the Web site www.webop.de. They then were instructed to concentrate on that chapter and to learn it by memorizing the videos, focusing on the procedural steps. After understanding the procedure, they additionally used the primer and learned the steps by heart. When they had finished this, they could choose using Webop, the primer, or both for the remainder of the training period. The total duration of the training time was 2 h.

##### Group 2 (practical training)

All the participants in this group stayed at the Pelvi-Trainer after they had completed the pretest. They then attended practical training for 2 h and conducted an average of two laparoscopic cholecystectomies during this time.

##### Group 3 (combination training: multimedia-based and practical training)

The participants in this group underwent multimedia-based training and practical training. After completing the pretest, they were led to the activity area (multimedia-based training). They received the same briefing as group 2, but their multimedia-based training lasted only 1 h. After the multimedia-based training, they were led to the Pelvi-Trainer, where they performed practical training for 1 h. On the average, they performed one laparoscopic cholecystectomy during that time. The entire training period lasted 2 h.

##### Group 4 (no training: control group)

All the participants randomized into this group were sent home after they had completed the pretest.

### Recording of data and evaluation

Both the pre- and posttesting were video recorded, encoded, and saved on DVDs. The DVDs were evaluated by blinded raters after all the experiments had been completed. The raters were given guidelines for the evaluation and intensively trained for evaluation of the videos. They learned the evaluation criteria by evaluating several example videos to ensure that the evaluations were consistent and correct. Calibration of the raters was performed by collective evaluation of 40 videos to ensure a high interrater reliability.

The main evaluation criterion was the difference in the objective structured assessment of technical skills (OSATS) score between the pre- and posttest (ΔOSATS). The OSATS is a tool for assessing practical skills [15] that integrates different assessment systems. It was modified for this study, as shown by Immenroth et al. [13].

The OSATS is characterized by the task-specific checklist that judges the specific procedural steps and the global rating scale as an overall performance evaluation. The task-specific checklist consists of 12 procedural steps, which are scored as correctly (1) or incorrectly (0) performed. The global rating scale considers five different surgical criteria, each scored 1 (least) to 5 (best). In the task-specific checklist, a maximum of 12 points can be achieved, and in the global rating scale, 25 points can be achieved (Table 2).

**Table 2:** Objective structured assessment of technical skills (OSATS)

Task-specific checklist
1. Placement of trocars
2. Exploration of the liver and display of the anatomic landmarks
3. Fixation of the infundibulum
4. Incision of the peritoneal layer on the infundibulum
5. Exposure of the cystic duct or cystic artery
6. Clipping of the cystic duct
7. Cutting of the cystic duct
8. Clipping of the cystic artery
9. Cutting of the cystic artery
10. Subserous shelling out of the gallbladder
11. Inspection of the liver bed
12. Recovering of the gallbladder in the salvage bag
Global rating scale
• Respect for tissue
• Time and motion
• Handling of instruments
• Flow of motion
• Knowledge of procedure

Videos were assigned to the raters by lot to ensure that each rater received the same number of videos from each group. Pre- and posttest videos of each participant were evaluated by the same rater. Raters were blinded to the participant, to the intervention group, and to the pre- or posttest.

### Sample size

Based on the results reported by Immenroth et al. [13], we calculated that recruitment of at least 60 participants would provide sufficient power (80 %) to detect an intergroup difference of 0.5 OSATS points at a significance level of 5 % [16]. We anticipated a standard deviation of 0.6 for OSATS change scores. We added another 15 % to compensate for potential problems such as missing posttests or technical problems with video recording. A total of 70 participants completed the study and were analyzed.

### Statistical analysis

**Fig. 3:**
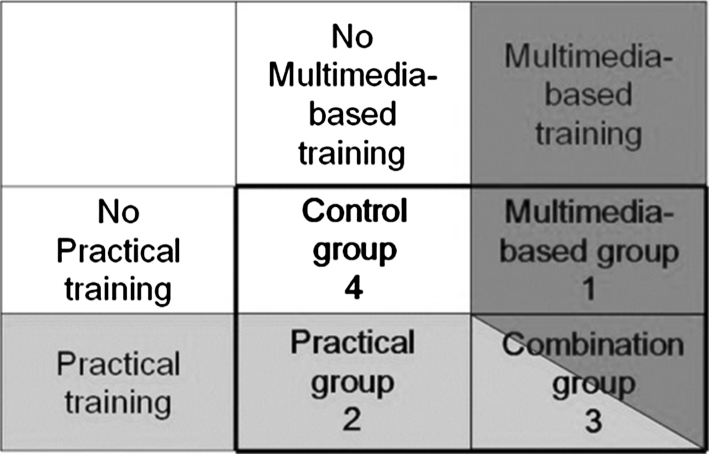
Factorial study design

All data were entered into a database. The OSATS and ΔOSATS data were approximately normally distributed. Therefore, intragroup comparisons of posttest and pretest measurements could be performed with an independently paired Student’s *t-*test, as required. Data were analyzed with SPSS (version 12.0). Based on the factorial study design (Fig. 3), the following two between-group comparisons were considered:

1. Effect of multimedia-based training (group 1 + 3 vs 4 + 2)
2. Effect of practical training (group 2 + 3 vs 4 + 1).

Statistical analyses for differences between the groups in terms of ΔOSATS were performed in two steps:

1. Analysis of variance (ANOVA) across all four groups (*p* < 0.05)
2. In the case of a significant result, the effect of multimedia-based training (group 1 + 3 vs 4 + 2) was compared with the effect of practical training (group 2 + 3 vs 4 + 1) (*p* < 0.05 each).

The intragroup effect of training was evaluated using the paired *t*-test to compare pre- and posttest results. To prevent the statistical error of multiple testing, levels of significance were adjusted according to Bonferroni–Holm.

## Results

### Study participants

The study enrolled 70 participants, with 18 participants randomized to the multimedia-based training group, 17 to the practical training group, 18 to the combination group, and 17 to the control group. The groups were homogeneous in terms of age, sex, and practical experience. Surgical fellows and students were equally distributed (Table 3).

**Table 3:** Baseline data of participants

	Multimedia-based training (*n* = 180)	Practical training (*n* = 17)	Combination training (*n* = 18)	Control group (*n* = 17)
Sex (female/male)	9/9	10/7	8/10	10/7
Age (years)	29 ± 3.6	27 ± 2.8	29 ± 3.9	28 ± 3.5
MD/student	13/5	9/8	11/7	8/9
No. of laparoscopic surgeries^a^	17/1	16/1	16/2	17/0

^a^Self-performed laparoscopic surgeries; see question 7 of the questionnaire; the answers were given in a range of 1–3 and 4–6 (e.g., in the practical training group, 16 participants had an experience of 1–3 laparoscopic surgeries and 1 participant had an experience of 4–6 laparoscopic surgeries

### Pre- and posttest

**Fig. 4:**
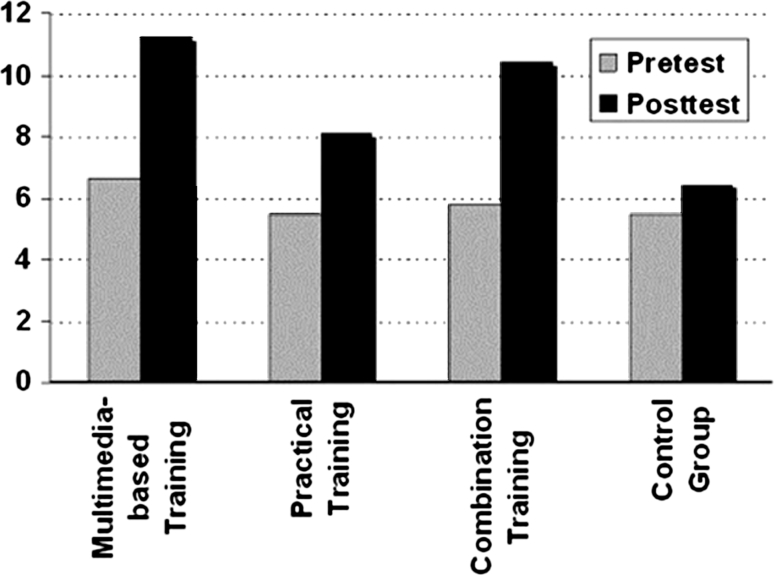
Pre- and posttest objective structured assessment of technical skills (OSATS) values

The pretest results for all the groups were comparable and homogeneous (Table 4; Fig. 4). There were no significant differences.

**Table 4:** Objective structured assessment of technical skills (OSATS) results

OSATS	Test	Multimedia-based training (*n* = 18)	Practical training (*n* = 17)	Combination training (*n* = 18)	Control group (*n* = 17)
Task-specific checklist	Pretest	6.6 ± 2.8	5.5 ± 3.7	5.8 ± 3.3	5.5 ± 2.8
	Posttest	11.2 ± 1.4	8.0 ± 3.2	10.4 ± 2.0	6.3 ± 3.2
	ΔOSATS	4.7 ± 3.3 (*p* < 0.001)^a^	2.5 ± 4.3 (*p* = 0.028)^b^	4.6 ± 3.5 (*p* < 0.001)^a^	0.8 ± 2.9 (*p* = 0.294)^b^
Global rating scale	Pretest	13.6 ± 5.1	11.9 ± 4.5	12.5 ± 5.9	12.1 ± 4.0
	Posttest	20.6 ± 3.1	15.9 ± 3.5	20.4 ± 3.4	13.9 ± 3.7
	ΔOSATS	6.9 ± 5.4 (*p* < 0.001)^a^	4.1 ± 4.1 (*p* < 0.001)^a^	7.9 ± 6.4 (*p* < 0.001)^a^	1.9 ± 4.4 (*p* = 0.100)^b^

^a^Statistically significant

^b^Not statistically significant

The follow-up test values showed significant differences among the intervention groups in terms of the task-specific checklist that ranged from 6.3 ± 3.2 (control group) to 11.2 ± 1.4 (multimedia-based group). The best values were achieved by the multimedia-based training group and the combination training group (Table 4; Fig. 4). Augmentation of the OSATS scores was higher in the groups undergoing multimedia-based training (multimedia-based training and combination training) than in the practical training group for all the procedural steps except steps 1 and 12, in which the practical training group reached the same result as the multimedia-based training group, and the combination group was even behind them in step 12 (Table 5).

**Table 5:** Results of the objective structured assessment of technical skills (OSATS) task-specific checklist (detail)

OSATS	Multimedia-based training	Practical training	Combination training	Control group
Task-specific checklist^a^	(*n* = 18)	(*n* = 17)	(*n* = 18)	(*n* = 17)
1. Placement of trocars	100→100	88→100	89→100	88→94
2. Exploration of the liver and display of the anatomic landmarks	94→100	88→82	89→100	82→64
3. Fixation of the infundibulum	50→100	47→82	56→100	47→71
4. Incision of the peritoneal layer on the infundibulum	44→100	47→82	44→100	17→70
5. Exposure of the cystic duct and cystic artery	33→100	23→58	28→72	17→23
6. Clipping of the cystic duct	61→100	41→59	39→89	53→53
7. Cutting of the cystic duct	56→100	35→70	44→89	53→47
8. Clipping of the cystic artery	17→78	23→41	22→66	17→17
9. Cutting the cystic artery	17→78	23→35	22→66	17→17
10. Subserous shelling out of the gallbladder	88→94	47→71	55→94	53→59
11. Inspection of the liver bed	39→89	23→35	33→89	41→23
12. Recovery of the gallbladder in the salvage bag	55→83	59→83	61→78	64→88

^a^Task-specific checklist data for each procedural step for all four groups: the first number is the pretest value and the number after the arrow is the posttest value

### ΔOSATS

The main interest of the study was the augmentation of the OSATS score after the training (ΔOSATS). The ΔOSATS score was highest in the multimedia-based training group (4.7 ± 3.3), with the practical training group achieving 2.5 ± 4.3, the combination training group achieving 4.6 ± 3.5, and the control group achieving 0.8 ± 2.9 in the task-specific checklist (Table 4). Subgroup analyses confirmed that these training effects were similar between physicians and last-year medical students.

### Effect of multimedia-based training

A total of 36 participants underwent multimedia-based training (multimedia-based training and combination training). They reached a ΔOSATS score of 4.6, whereas the participants without multimedia-based training (practical training and control groups; *n* = 34) reached a ΔOSATS score of 1.7. With a *p* value of 0.001, the effect of multimedia-based training was significant.

### Effect of practical training

A total of 35 participants underwent practical training (practical training and combination training). They reached a ΔOSATS score of 3.6. The participants without practical training (multimedia-based training and control groups; *n* = 35) reached a ΔOSATS of score 2.8. Practical training did not have a significant effect on surgical performance (*p* = 0.38).

## Discussion

The main interest of the study was to compare the effect of multimedia-based training with the effect of practical training on the surgical performance of surgical novices. The current study confirmed that multimedia-based training improved surgical performance of laparoscopic cholecystectomy in a Pelvi-Trainer significantly when used alone or as combination training. The participants undergoing practical training alone did not achieve similarly improved results.

The groups had no major differences in terms of demographic or baseline test data, although the multimedia-based training group had the best results in the baseline tests. Because randomization was adequately concealed, this effect can be seen as unpredictable coincidence. Because the primary outcome criterion was the difference between posttest (follow-up) and pretest (baseline) results, ΔOSATS was not affected by these higher baseline results. Quite the contrary, to achieve higher ΔOSATS results, posttest values had to be even higher because pretest values were subtracted.

During the experimental phase, the participants could contact other people including their co-participants. To reduce bias of possible effects from these contacts, we performed the randomization after the pretest. Talking or changing intervention groups by the participants could thus be avoided.

Introduction to the Pelvi-Trainers was identical for all the participants because randomization took place after the introduction. The introduction was always performed by the same team members, who followed a written guideline containing the information they were allowed to present. Even if the introduction varied by different team members, this could not influence the results because one experimental group always consisted of one participant from each intervention group based on the study design, eliminating subsequent effects. Effects of the different camera assistants on the OSATS results also could be eliminated because they were blinded (in pre- and posttest) and not allowed to move the camera without the participant’s command.

Before leaving the lab, all the participants had to sign an agreement that they would not take advantage of outside opportunities to improve their knowledge about laparoscopic cholecystectomy. Whether the participants kept their promise or not cannot be proven, but at least consecutive errors were avoided.

Immenroth et al. [13] did not find any major differences in the global rating scale comparing mental training with practical training but rather in the task-specific checklist. Referring to these results, we defined our aim criterion as the difference between the follow-up test after intervention (training) and the baseline test before intervention, measured in terms of the OSATS criteria of the task-specific checklist: ΔOSATS. A secondary outcome criterion was the ΔOSATS of the global rating scale.

Findings have shown OSATS to be a feasible measuring tool [15] that can reliably and validly assess surgical skills [17]. The task-specific checklist determines the ability of the participant to perform the individual steps in the sequence of a laparoscopic cholecystectomy in the Pelvi-Trainer. Hence, it is a combination of cognitive and practical tasks. Comparing a task-specific checklist, Immenroth et al. [13] assumed that the task-specific checklist evaluated the more cognitive components of a surgical procedure. The global rating scale should consider the motor skills [13]. Martin et al. [17] even declared global rating scales to be a better method of assessment than task-specific checklists [17]. Most of the authors, having used OSATS in their studies, do not distinguish between the task-specific checklist and the global rating scale in terms of one being a better method than the other.

Our findings support the aforementioned opinion. Groups undergoing multimedia-based training had the best results in terms of the task-specific checklist and the global rating scale compared with the practical training group. Because cognitive comprehension (learning the individual procedural steps) is elicited in the task-specific checklist, it is not too surprising that the multimedia-based training group showed the best results in this assessment, although the participants were asked to transform the theoretical knowledge they had learned with multimedia-based training to practical performance in the Pelvi-Trainer.

Except the control group, all the intervention groups showed significant improvement in the global rating scale (*p* ≤ 0.001). An amazing result was that both groups undergoing multimedia-based training (multimedia-based and combination training) were better (respectively 6.94 ± 5.35 and 7.94 ± 6.35) than the practical training group (4.06 ± 4.09) in the global rating scale (see data tables) despite the assumption that the practical training group had more practical experience concerning the skills determined by global rating scale after the intervention (Table 6).

**Table 6:** Results of the objective structured assessment of technical skills (OSATS) global rating scale (detail)

ΔOSATS	Multimedia-based training (*n* = 18)	Practical training (*n* = 17)	Combination training (*n* = 18)	Control group (*n* = 17)
Global rating scale^a^				
Respect for tissue	1.2 ± 1.7	0.5 ± 1.2	1.3 ± 1.8	0.1 ± 1.3
Time and motion	1.3 ± 1.2	0.9 ± 1.0	1.4 ± 1.3	0.7 ± 1.1
Handling of instruments	1.4 ± 1.2	0.7 ± 1.1	1.7 ± 1.4	0.2 ± 1.4
Flow of motion	1.2 ± 1.1	1.0 ± 1.1	1.6 ± 1.3	0.3 ± 1.2
Knowledge of procedure	1.8 ± 1.2	0.9 ± 1.4	1.9 ± 1.4	0.6 ± 1.0

^a^Global rating scale data for each rated detail for all four groups as mean ± standard deviation. Shown is the improvement from pre- to posttest value: Δ

Compared with the findings of Immenroth et al. [13] that mental training showed more effect on the cognitive aspects of the procedure, we showed that multimedia-based training improves not only cognitive skills but also simple motor skills more than practical training alone. Apparently multimedia-based training not only teaches cognitive skills but also improves practical skills in a way that imparts the sense of tissue, the handling of instruments, and last but not least, the procedure itself. In a sense, this comprises the way surgery was taught in former days, with the surgical trainee adopting skills from the surgical teacher via a “see one, do one, teach one” approach. However, this way of teaching does not fit in our daily practice, and many physicians regard this training as insufficient [18]. The time spent in the OR teaching raises enormous and inappropriate costs if this is the only venue of teaching [15]. Additionally, this type of teaching is insufficient because trainees learn by practicing on real patients, and the residents feel inadequately trained to perform procedures by themselves [19]. This consequent uncertainty leads to mistakes [20, 21].

Multimedia-based training offers a solution for these problems. The way the surgeries are presented in combination with videos allows the surgeon to “watch” the surgery and adopt the ways of the experienced surgeon. After undergoing multimedia-based training, the surgeon probably will feel better trained and more secure in the procedure itself, possibly avoiding potential mistakes. Before practicing on patients, the surgeon has already gathered some knowledge and will not use expensive time in the OR for practice.

As one of five Internet platforms, www.webop.de provides surgical know-how of general and abdominal surgery that has been identified by a former review including 31 criteria for the fields of “content presentation,” “infrastructure,” and “evaluation” [6]. We chose www.webop.de for our study to provide content in a uniform educational manner with the focus on easy understanding for the user. Comparable evaluations are unknown to us. It will be a task for future studies to compare the learning effects of the different platforms.

Although blind and randomized controlled trials are the best way to show possible differences in interventions, the results cannot be transferred to real-life teaching of young surgeons completely without considering the limitations of the study. Although it could be assumed that the relaxed atmosphere in the lab cannot be compared with the tense atmosphere in the OR, various studies have shown that skills acquired by simulation-based training seem to be transferrable to the setting in an OR [22]. Findings have shown that even in simulations, surgeons experience stress levels, especially when undergoing crisis-simulation in virtual trainers [21], whereas general statements concerning the influence of stress on the surgical performance cannot be made due to lack of homogeneous studies [20]. Nevertheless, the stress factor of operating on an organ model of a dead liver rather than a living patient may not be comparable. It therefore may lead to better results than could have been achieved in the OR.

Contrariwise, the relaxed atmosphere in the lab may have led to carelessness and impreciseness of some participants due to the lack of any vital consequences. However, some of the participants may have acted better and some may have acted worse because of the lab atmosphere, so the lab environment may not have had any consecutive effect.

In 2002, Seymour et al. [23] showed that VR simulation significantly improves OR performance of residents when carrying out a laparoscopic cholecystectomy. These data were achieved in a small study including 16 surgical residents. A review in 2008 urged caution in seeing the positive data of similar studies without looking beyond and considered the VR-to-OR skills transfer study model as a means of demonstrating the superiority of VR training activity over that of the simulator itself [24]. All in all, it can be said that training improves OR performance compared with no training. But it is a task of the future to compare the value of the different training methods and their effect on OR performance. Therefore, we do not know whether our positive results with multimedia-based training can be transferred to the OR.

## Conclusion

Multimedia-based training significantly improved surgical performance of a laparoscopic cholecystectomy in a Pelvi-Trainer. Statistically, practical training did not significantly improve surgical performance. In conclusion, multimedia-based training is a low-cost, always-available means of education that should not replace face-to-face teaching. It can be seen as a reasonable additional tool to be included in surgical curricula because it leads to improvement in surgical performance. The benefit of learning with the multimedia-based Internet platform www.webop.de can be defined as proven. In addition, changes in the World Wide Web, with a shift to more social-networking activity in education and Web-based delivery to small, ubiquitous portable devices will increase opportunities for surgical e-learning [25]. The use of surgical online platforms such as www.webop.de will therefore become even more interesting.
